# Probing mechanobiological role of filamin A in migration and invasion of human U87 glioblastoma cells using submicron soft pillars

**DOI:** 10.1186/s40580-021-00267-6

**Published:** 2021-07-02

**Authors:** Abdurazak Aman Ketebo, Chanyong Park, Jaewon Kim, Myeongjun Jun, Sungsu Park

**Affiliations:** 1 Department of Mechanical Engineering, Sungkyunkwan University (SKKU), 16419 Suwon, Korea; 2 Department of Biomedical Engineering, Sungkyunkwan University (SKKU), 16419 Suwon, Korea; 3 Institute of Quantum Biophysics (IQB), Sungkyunkwan University (SKKU), 16419 Suwon, Korea; 4 School of Mechanical Engineering, Sungkyunkwan University (SKKU), 2066 Seobu-ro, 16419 Suwon, Korea

**Keywords:** Filamin A, Traction force, Rigidity sensing, Cell adhesion, Motility, Filopodia

## Abstract

Filamin A (FLNa) belongs to an actin-binding protein family in binding and cross-linking actin filaments into a three-dimensional structure. However, little attention has been given to its mechanobiological role in cancer cells. Here, we quantitatively investigated the role of FLNa by analyzing the following parameters in negative control (NC) and FLNa-knockdown (KD) U87 glioma cells using submicron pillars (900 nm diameter and 2 μm height): traction force (TF), rigidity sensing ability, cell aspect ratio, migration speed, and invasiveness. During the initial phase of cell adhesion (< 1 h), FLNa-KD cells polarized more slowly than did NC cells, which can be explained by the loss of rigidity sensing in FLNa-KD cells. The higher motility of FLNa-KD cells relative to NC cells can be explained by the high TF exerted by FLNa-KD cells when compared to NC cells, while the higher invasiveness of FLNa-KD cells relative to NC cells can be explained by a greater number of filopodia in FLNa-KD cells than in NC cells. Our results suggest that FLNa plays important roles in suppressing motility and invasiveness of U87 cells.

**Supplementary Information:**

The online version contains supplementary material available at 10.1186/s40580-021-00267-6.

## Introduction

Metastatic cells are highly motile and undergo cell morphology changes that facilitate penetration through the three-dimensional (3D) structures of the extracellular matrix (ECM). During invasion, metastatic cells form thin cellular protrusions called filopodia [1, 2] in the direction of movement to form focal adhesions (FAs) [3]. Filopodia play an important role in cell migration by sensing and forming initial contact with ECM. The primary function of FA is to transmit traction forces (TFs) through actin filaments and attach cells to the ECM [4]. In addition, metastatic cells must be able to attach and survive in different or secondary sites with ECM of variable stiffness [5, 6]. However, the physical mechanisms by which metastatic cancer cells move through 3D structures of ECM and survive in ECM with variable stiffness are largely unknown. Understanding these mechanisms ultimately requires technology that can accurately quantify the TFs and rigidity sensing ability of cancer cells.

**Fig. 1:**
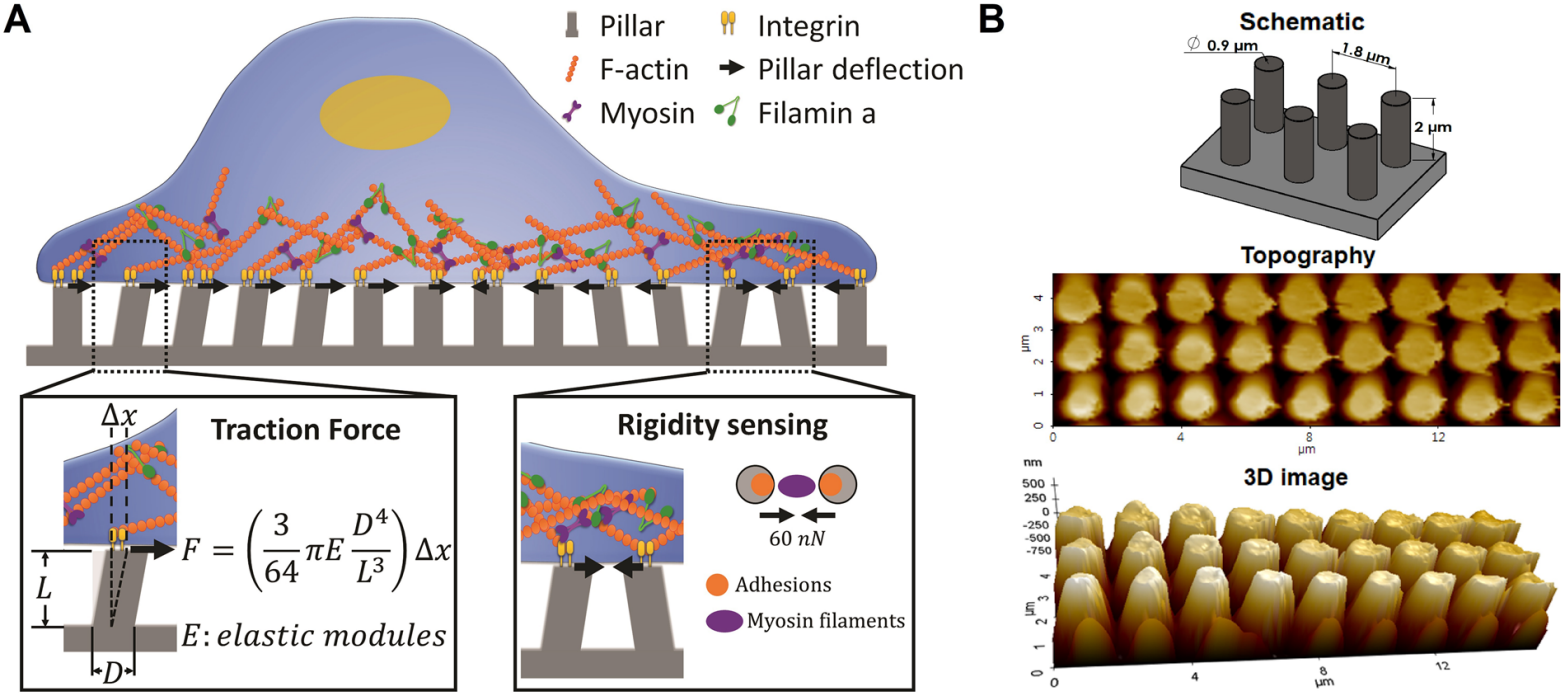
**A** Schematic describing traction forces (TFs) and rigidity sensing in a cell. F, D, L and E represent TF, the diameter, length, and Young’s modulus of PDMS, respectively. *∆x*; the displacement of the pillar. **B** Schematic and AFM (2D and 3D) topographical images of the pillar arrays. The AFM image of the pillar array was taken in ambient conditions in a contact mode. It shows that the diameter of pillars and center-to-center (c-t-c) distance between pillars are 900 nm and 1.8 μm respectively

TF microscopy that uses micron or submicron pillars [7–9] made of the elastomeric polymer polydimethylsiloxane (PDMS) can be used to quantify TFs and rigidity sensing of cells (Fig. 1). Tangential tension by a cell deflects the pillar. The degree of deflection and the bending stiffness of the pillar are used to quantify TFs exerted by the cell [10]. Recent studies [11] showed that cells sense the rigidity of ECM by producing local contractions through actin-myosin interactions. These local contractions can be detected by observing the deflection of two neighboring pillars towards each other (Fig. 1A). TF microscopy showed that cancerous cells generated higher TFs than non-cancerous cells [12]. However, it has not been determined whether mutations in cancer cells that increase metastasis, motility and invasion can also increase TFs.

Filamin A (FLNa) is a V-shaped actin-binding protein that is abundantly expressed in human cells (Fig. 1A). It binds and cross-links cortical actin filaments into a 3D structure [13] and mediates the conversion of mechanical forces into biochemical signals [14]. It also links integrins to the actin filaments [15, 16]. Thus, it integrates the cellular architecture and signaling that are essential for cell locomotion and development [17]. FLNa expression or suppression may facilitate or inhibit cancer invasion depending on the cell type, FLNa expression levels, and FLNa’s interactions with other proteins. For example, the migration speed of FLNa-deficient melanoma and breast cancer cells were significantly lower than wild type cells [18]. In contrast, FLNa-knockdown (FLNa-KD) increased the frequency of multifocal tumors of glioma cell lines in immunodeficient mice, and it increased the migration speeds of other glioma cell lines (A172 and LN-229) in vitro [19]. The contradictory results from these studies show that the role of FLNa in cancer metastasis and invasion remains elusive [20].

Here, we evaluated the role of FLNa in cancer invasion by measuring TFs and rigidity sensing of negative control (NC) and FLNa-KD glioma (U87) cells. We used submicron pillars (diameter, 900 nm; height, 2 μm; and center-to-center (c-t-c) distance between pillars, 1.8 μm; bending stiffness, k = 24.2 nN/µm) (Fig. 1B). The migratory and invasive behaviors of these cells were studied in conventional motility and invasion assays to demonstrate that mechanobiological results can be used as biomarkers for cancer metastasis.

## Experimental

### Cell culture and transfection

The Uppsala 87 malignant glioma (U87) cell line was purchased from American Type Culture Collection (ATCC, Manassas, VA). Cells were incubated in Gibco 1× minimum essential medium (Thermo Fisher Scientific) containing 10 % fetal bovine serum (FBS) (Gibco®, Thermo Fisher Scientific) and 100 units/ml penicillin (Sigma-Aldrich) under humid conditions at 37˚C in 5 % CO_2_. Cells were transfected with either AccuTarget™ FLNa small interfering RNA (siRNA) oligonucleotide (target sequence: 5’-AAGATGGATTGCCAGGAGTG-3’) or AccuTarget™ negative control (NC) siRNA from Bioneer Co. (Daejeon, Korea) according to the manufacturer’s instructions.

### Western blot analysis

The total extract of NC and FLNa-KD cells was prepared using radioimmunoprecipitation assay buffer (LPS solution, Daejeon, Korea) and protease inhibitor cocktail (Roche). Anti-FLNa antibody from rabbit (Abcam) and anti-rabbit antibody conjugated with horseradish peroxidase were used as primary and secondary antibodies to visualize FLNa. Glyceraldehyde 3-phosphate dehydrogenase (GAPDH) was used as control. The image of the blot was captured using a chemiluminescent imaging system (Atto, Daejeon, Korea) and analyzed using ImageJ (NIH, Bethesda, MD).

### Pillar fabrication

Photolithography with reactive ion etching (RIE) was used to fabricate a mold of linearly arranged arrays of holes (900 nm, diameter; 2 μm, depth; and 1.8 μm, c-t-c distance between holes) over 5-inch silicon wafers. PDMS Sylgrad® 184 silicon elastomeric base (Dow Corning Co.) [21] was mixed with its curing agent at either a 5:1 or 10:1 (w/w) ratio to obtain PDMS elastic modules of 4 and 2 MPa [22] and the mixture was degassed for 15 min. Next, the degassed mixture was spin-coated over the mold at 500 rpm for 10 s and subsequently at 1,800 rpm for 30 s before being degassed for 30 min to remove any trapped bubbles from the coated layer. The coated mold was cured at 80 °C for 4 h. Next, the PDMS layer was peeled off from the mold. The bending stiffness (*k*) of pillars made of PDMS at 5:1 and 10:1 mixing ratios of the elastomeric base and its curing agent was calculated to be 48.3 and 24.2 nN/µm using the following equation based on the Euler-Bernoulli beam theory: [23]

$$k=\frac{3}{64} \pi E\frac{{D}^{4}}{{L}^{3}}$$

where *D*, *L*, and *E* represent the diameter, length, and Young’s modulus of PDMS, respectively. The pillar array was probed by an atomic force microscope (AFM) using XE-7 (Park Systems, Suwon, Korea). Imaging under ambient conditions was conducted in a contact mode with PPP-CONTSCR cantilever (NANOSENSORS™, Neuchatel, Switzerland) with a nominal force constant of 0.2 N/m and a nominal resonance frequency of 25 kHz.

### TF measurements

To observe the deflection of pillars by cells, images of pillars with cells were obtained at 1 and 6 h in a live cell chamber (Live Cell Instrument, Seoul, Korea) at 37 °C with 5 % CO_2_ using a DeltaVision microscope (GE healthcare) equipped with a CoolSnap HQ^2^ camera (Photometrics, Tucson, AZ). PillarTracker version 1.1.3, which was obtained from the Mechanobiology Institute (MBI) in Singapore, was used to measure pillar deflection. PillarTracker applies a reconstruction algorithm to create a perfect grid for measuring pillar deflection. Pillars outside each cell were used as the reference corresponding to the zero-force position. The deflection value was multiplied by the bending stiffness of the pillar to determine the pillar’s traction force (Fig. 1A).

### Rigidity sensing

The rigidity sensing ability of a cell was determined by calculating the directionality parameter (*γ*) during the initial phase of spreading (< 30 min) at the leading edges (an area of 34.5 µm^2^) of the boundary of the cell as a measure of local contractions [24]. *γ* is calculated as the sum of the force vectors of pillars divided by the sum of their magnitudes. For this purpose, cell images taken within 30 min of cell substrate contact were used for γ calculation. Average *γ* values of each type of cells were obtained by calculating fifty frames of six different cells.

### Fluorescent imaging of F-actin

Cells were incubated on fibronectin-coated pillars in an incubator containing 5 % CO_2_ at 37 °C for 1 and 6 h. The cells were washed with phosphate-buffered saline (PBS) (pH 7.4) and fixed with 4 % paraformaldehyde in PBS at room temperature (RT) for 10 min. Cells were permeabilized with 0.5 % triton X-100 in PBS at RT for 5 min. F-actin was first stained with rhodamine phalloidin (RP) (Sigma-Aldrich) (1:1,000) in PBS at RT for 30 min, and the nucleus was subsequently stained with 0.2 mg/mL of 4’,6-diamidino-2-phenylindole (DAPI) (Sigma-Aldrich) in PBS at RT for 15 min.

### Measurement of cell aspect ratio on pillars

Fluorescent images of cells stained with RP and DAPI were obtained. Cell shape was represented as an equivalent elliptical shape using ImageJ. The aspect ratio, which was used as a measure of cell polarity, was determined by dividing the major axis of the ellipse by the minor axis.

### Immunostaining of PXN and fascin

For FA analysis, cells that were incubated on fibronectin-coated glass slides in an incubator containing 5 % CO_2_ at 37 °C for 1 and 6 h were washed, fixed, and permeabilized as described above. The cells were immunostained with rabbit anti-PXN antibody (Abcam) for 1 h. Next, they were incubated with anti-rabbit antibody conjugated with Alexa Fluor® 488 (Thermo Fisher Scientific) for 45 min. Finally, fluorescent images were taken using a DeltaVision microscope.

To image filopodia, cells that were incubated on fibronectin-coated pillars in an incubator containing 5 % CO_2_ at 37 °C were washed, fixed, and permeabilized as described above. They were immunostained with rabbit anti-fascin primary antibody (Abcam) for 1 h. Next, they were incubated with anti-rabbit antibodies conjugated with Alexa Fluor® 488 for 45 min. Finally, fluorescent images were taken using a DeltaVision microscope (GE Healthcare).

### Cell motility experiment and invasion assay

Pillars were coated with fibronectin for 1 h before seeding cells. Cells were seeded at a density of 3000 cells per sample. They were tracked every 5 min for 6 h using a JuLi™ Br live cell movie analyzer (NanoEntek, Inc., Seoul, Korea). The migration speed of NC and FLNa-KD cells (n = 10) were calculated after tracking the movement of the cell using ImageJ. An invasion assay was performed using modified Boyden Chambers BioCoat™ Matrigel® Invasion Chamber (Corning) comprised of Matrigel membrane filter inserts with 8 μm pores in a 24-well tissue culture plate [25]. A total of 2 × 10^4^ cells were mixed with minimum essential medium (MEM) with 20 % FBS and seeded onto the top of the chamber, and the bottom chamber was filled with MEM containing 20 % FBS as a chemoattractant. The cells were incubated at 5 % CO_2_ and 37 °C for 24 h. Non-invading cells were removed from the upper membrane using a cotton swab, and invasive cells were fixed with 4 % paraformaldehyde and stained with toluidine blue (Sigma-Aldrich). Stained cells were counted randomly from three locations on the membrane. The experiment was repeated at least three times.

### Statistical analysis and error correction

Throughout this study, all data were represented by the mean ± standard error of the mean of the samples. The student’s t-test was used to compare NC and FLNa-KD samples. A P-value of less than 0.05 was considered significant (*P < 0.05, ** P < 0.01, *** P < 0.001). In the TF experiments, the deflection errors of the pillars were reduced by subtracting the mean deflection of the empty pillars outside the cell boundary.

## Results and discussion

### Delayed cell spreading due to impairment in rigidity sensing

**Fig. 2:**
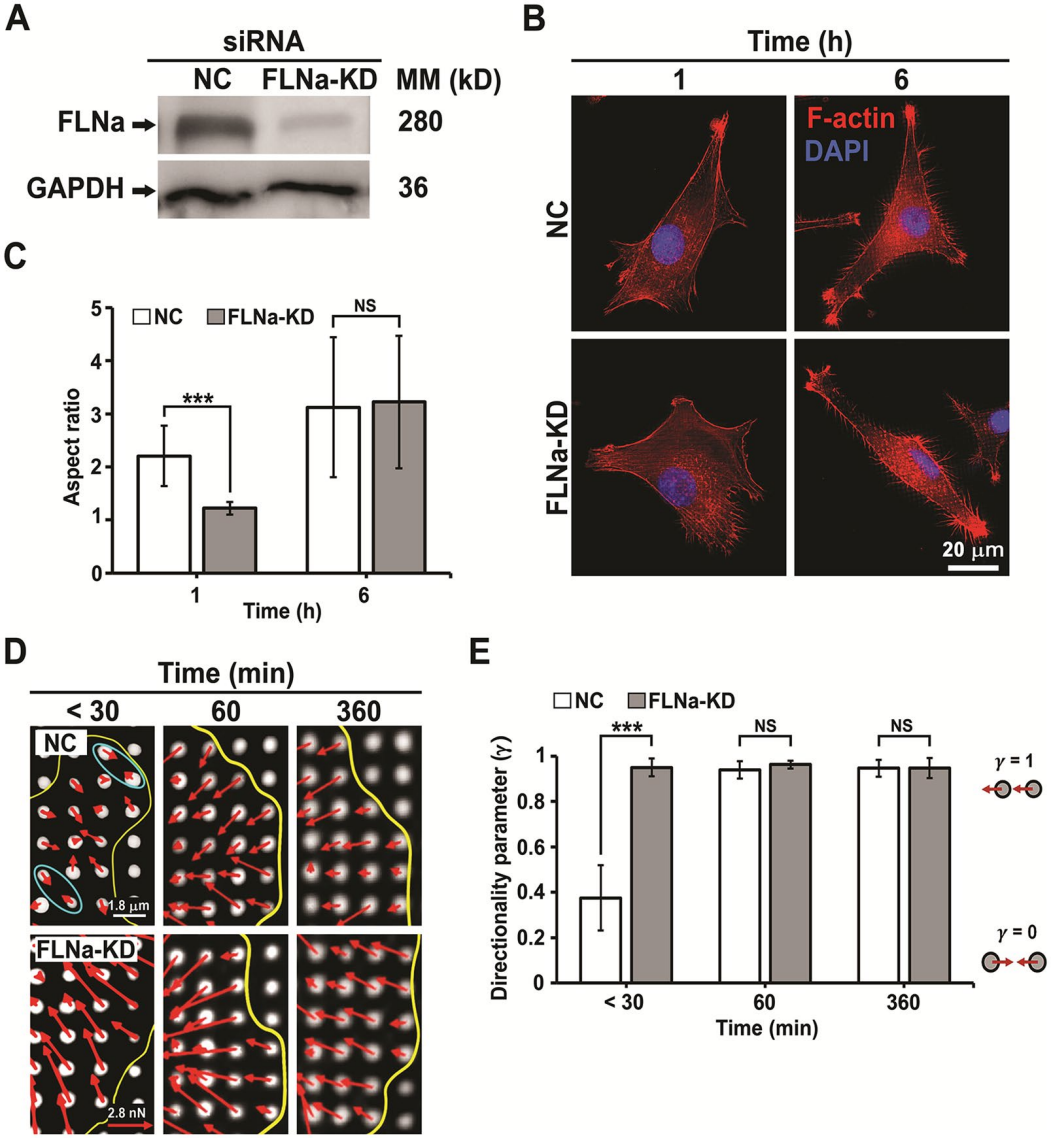
Cell spreading and rigidity sensing ability of NC and FLNa-KD cells on fibronectin-coated pillars (diameter, 900 nm; height, 2 μm; and pillar center-to-center distance, 1.8 μm; bending stiffness *k* = 24.2 nN/µm). **A** Western blot of FLNa. Control: GAPDH. **B** Fluorescence images of F-actin (RP: red) and the nuclei (DAPI: blue) of cells on fibronectin-coated pillars at 1 and 6 h. **C** Aspect ratio of cells. (n = 30 cells). **D** Deflection of pillars in NC and FLNa-KD cells near the edge (approximately 34.5 µm^2^) of cells at < 30, 60, and 360 min incubations. Local contractions in the NC cells are marked with cyan circles. The red arrows indicate pillar deflection. **E** Directionality parameters (*γ*) of the NC and FLNa-KD cells at < 30, 60, and 360 min incubations. *γ* is calculated as the sum of the force vectors of pillars divided by the sum of their magnitudes. *γ* is 1 when the force vectors of two neighboring pillars are parallel. *γ* is 0 when the force vectors of the pillars are locally contracted toward each other. Cell number n = 6. Error bar represents the mean ± standard deviation (SD). *** P < 0.001. Student’s t-test. *NS* Not significant

During initial cell adhesion, the leading edges of cells undergo periodic protrusion-retraction cycles to test ECM rigidity [26]. Cells control their spreading based on ECM rigidity [27]. To investigate the role of FLNa on cell spreading, the shape of NC and FLNa-KD cells incubated on fibronectin-coated pillars (Fig. 1B) for 1 and 6 h was observed with actin staining and analyzed by measuring their aspect ratio. Before incubation, FLNa knockdown was verified in cells using western blotting (Fig. 2A). At 1 h, FLNa-KD cells were relatively round and less polarized than NC cells (Fig. 2B), which is supported by the fact that the cell aspect ratio of the FLNa-KD cells was significantly lower than that of the NC cells at 1 h (P < 0.001). At 6 h, FLNa-KD cells were elongated to a similar extent as NC cells (Fig. 2B), which is supported by the fact that there was no significant difference in the aspect ratio between NC and FLNa-KD cells (Fig. 2C). These results suggest that FLNa-KD can delay cell spreading when cells contact ECM for a limited period (about 1 h) but the delayed effect of FLNa-KD in cell polarization diminished at 6 h.

Cells sense the rigidity of the substrate by undergoing local contractions. It occurs during the initial phase of cell spreading (< 30 min). Local contractions by cells occur through contractile units (CUs) consisting of cytoskeletal protein complexes, including tropomyosin, myosin II, integrin, and α-actinin (Fig. 1A) [28]. To understand why FLNa-KD cells delayed spreading on pillars (Fig. 2B), we investigated the effect of FLNa-KD on rigidity sensing by observing the deflection of pillars at the leading edges (an area of 34.5 µm^2^) of the boundary of NC and FLNa-KD cells at < 30 min, 60 min, and 360 min [29]. For < 30 min, locally contracted pillars were observed on the leading edges of the NC cells but not on the leading edges of FLNa-KD cells (Fig. 2D). At 60 and 360 min, all pillars were centripetally deflected in both NC and FLNa-KD cells. The deflected direction of pillars was further analyzed to determine the directionality parameter (*γ*). *γ* is 1 when the force vectors of two neighboring pillars are parallel. *γ* is 0 when the force vectors of the pillars are locally contracted toward each other. At < 30 min, *γ* of the NC cells was 0.4 ± 0.14 (mean ± standard deviation (SD)), indicating that they sensed the rigidity of the pillars (*k* = 24.2 nN/µm) (Fig. 2E). However, *γ* of the FLNa-KD cells was 0.9 ± 0.03 (mean ± SD) (Fig. 2E), indicating that they could not sense the rigidity of the pillars [29]. At 60 and 360 min, the *γ* values of both NC and FLNa-KD cells were about 0.9. This result shows that the local contraction was observed only during the initial phase of spreading for U87 cells with intact FLNa. Similarly, the local contraction by NC triple negative breast cancer (MDA-MB-231) cell was observed on pillars (*k* = 24.2 nN/µm) for < 30 min (Additional file 1: Figure S1A, B), demonstrating that the local contraction of the pillar is not dependent on the bending stiffness of pillars. CU proteins like FLNa and tropomyosin are required to exert local contractions in cells [30]. Most cancer cells including glioma cells are known to have decreased tropomyosin expression. Together with the previous report, our results imply that the NC U87 cells do not completely lose their rigidity sensing. Cells can grow on substrate with low rigidity even when their rigidity sensing is completely impaired [31]. The loss of rigidity sensing is advantageous for metastatic cell survival in different or secondary sites with variable stiffness of the ECM. In particular, FLNa plays important roles in stabilizing early adhesion sites through its direct binding to integrin. Thus, FLNa-KD might have delayed the recruitment of proteins to adhesion sites, including α-actinin, actin filaments, and tropomyosin [32]. CUs cannot form without these proteins. This may explain why locally contracted pillars were not observed in the FLNa-KD cells. Taken together, these data suggest that the delay in spreading in the FLNa-KD cells during the initial phase of cell spreading (Fig. 2B, C) could be due to the loss of rigidity sensing (Fig. 2D, E).

### High motility and traction force in FLNa-KD cells

**Fig. 3:**
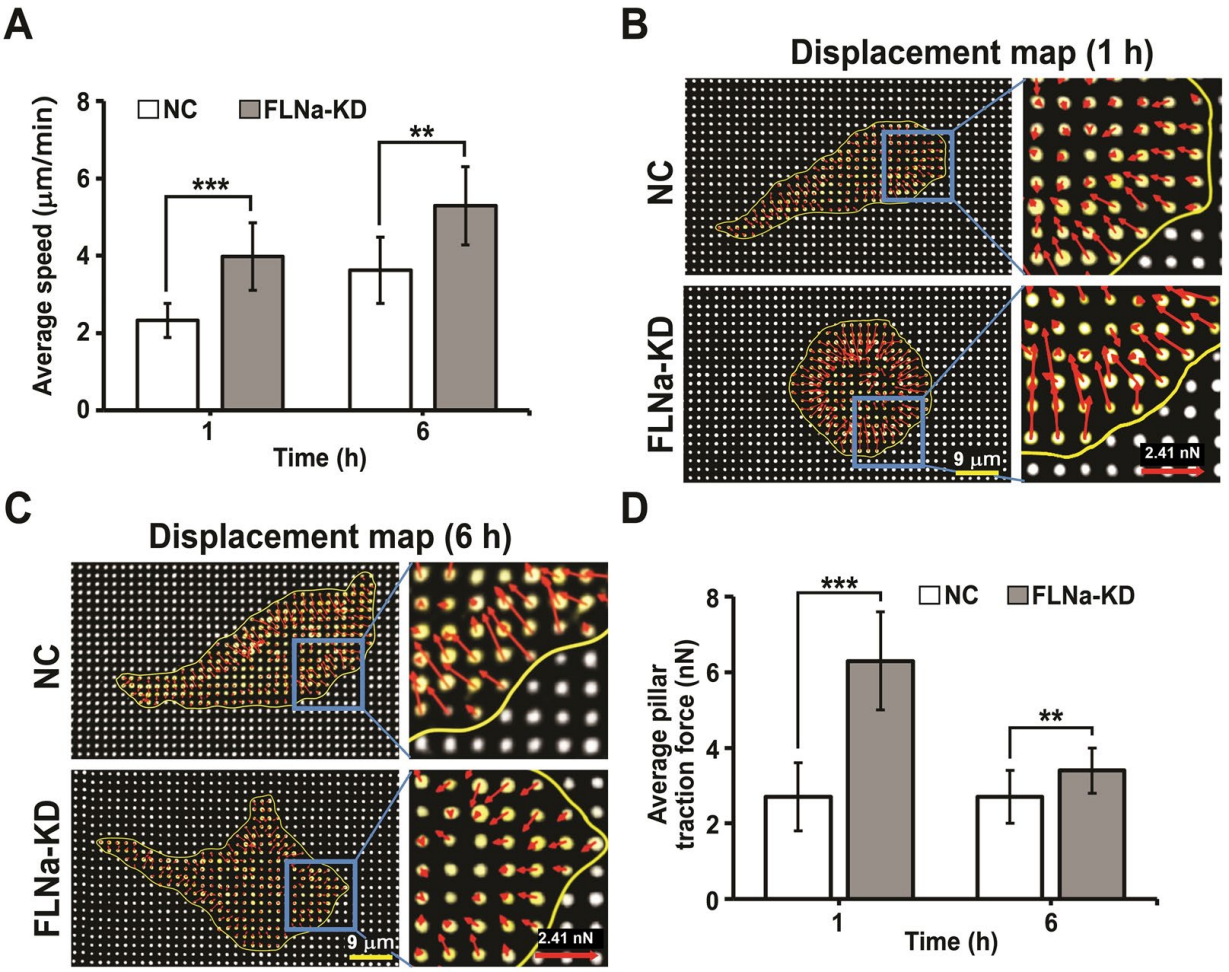
Motility and TFs of NC and FLNa-KD cells on fibronectin-coated pillars (**A**) Average speed of cells (n = 10 cells) at 1 and 6 h of incubation on the pillars. The representative deflection maps (**B**, **C**) of the NC and FLNa-KD cells on pillars at 1 h (**B**) and 6 h (**C**). The yellow line is the approximate boundary of the cells. The red arrow indicates the deflection magnitude and direction of each pillar. (**D**) Average TFs of the cells at 1 and 6 h. Number of samples, (n = 40 cells). Error bar represents the mean ± SD; ** P < 0.01, *** P < 0.001; Student’s t-test

Metastatic properties of cancer cells like high motility can be studied by quantifying individual cell motilities. Using fibronectin-coated pillars the motility of individual cell was monitored for 6 h. The FLNa-KD cells migrated faster than the NC cells at 1 and 6 h (Fig. 3A), indicating that FLNa-KD increased cell motility.

Cell mechanics and migration are known to be directly related [33–35]. TFs in FLNa-KD and NC cells were compared to quantitatively describe how FLNa-KD increased cell migration speed. TFs exerted by the FLNa-KD and NC cells were calculated using the deflection data of each pillar with the bending stiffness (*k* = 24.2 nN/µm). At both 1 and 6 h, the TFs of the FLNa-KD cells were significantly higher than the NC cells (Fig. 3D) (Additional file 2: Movie. S1 and Additional file 3: Movie. S2). A similar result was also observed in MDA-MD-231 cells (Additional file 1: Figure S2). Since FLNa competes with talin to bind with integrin [36, 37], talin binding to the integrin β tail increases when FLNa is impaired and thus enhances integrin activation and cell motility through FA turnover. These results suggest that FLNA-KD cells increase their motility by exerting high TFs.

### Effect of FLNa-KD on cell FA

**Fig. 4:**
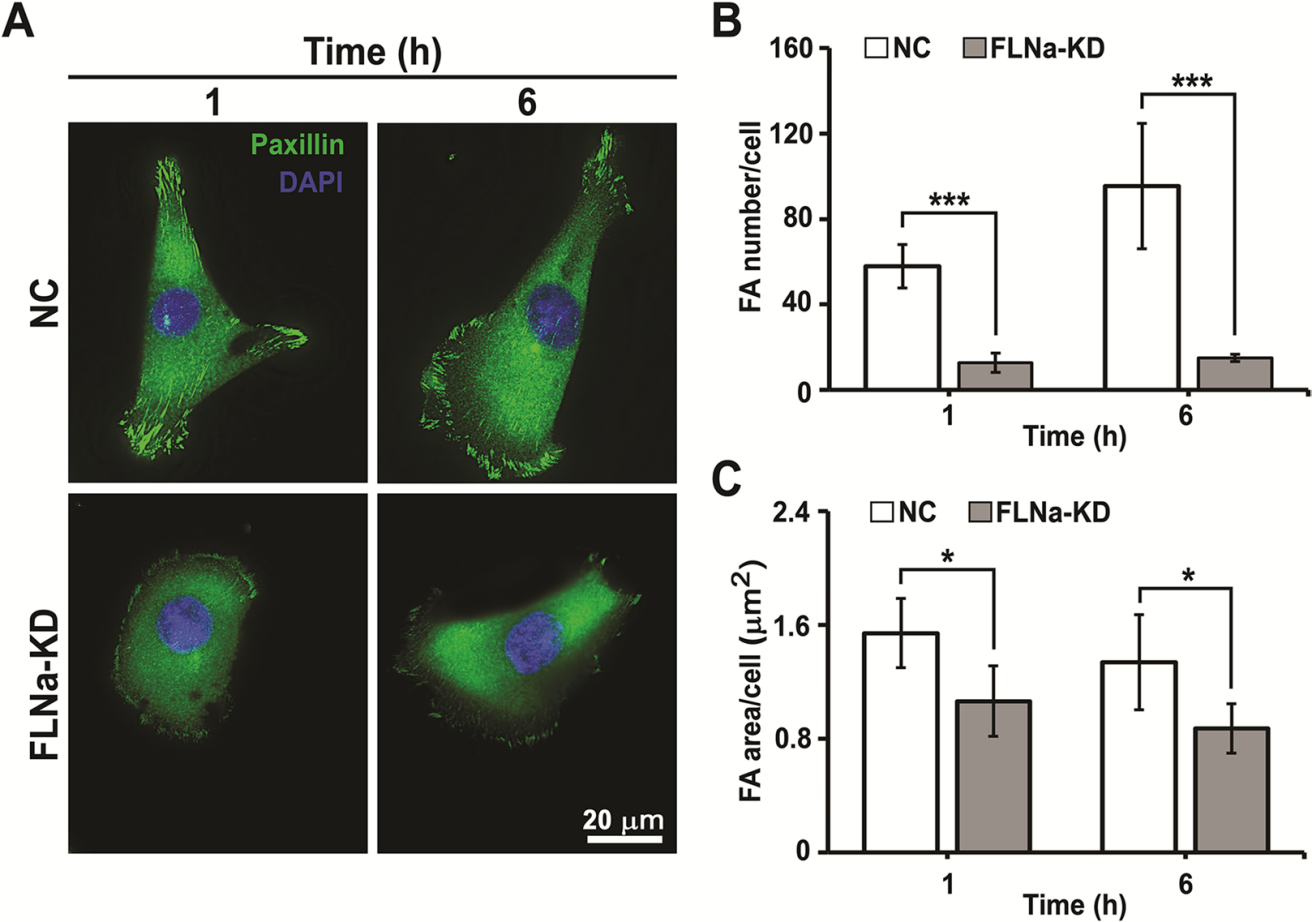
FA number and size of NC and FLNa-KD cells on glass slides coated with fibronectin at 1 and 6 h. **A** Immunostaining of paxillin (PXN, green) in cells. FA number (**B**) and size (**C**). Error bar represents the mean ± SD. (n = 6 cells). *P < 0.05; ***P < 0.001; Student’s t-test

FAs comprise several kinds of proteins, including filamin (FLNa) and paxillin (PXN), which perform different functions. For example, integrins are signaling proteins that link the ECM with the cell cytoskeleton [38]. PXN is an FA protein that controls cell spreading and motility [39]. FLNa is known to mediate the phosphorylation PXN in androgen-stimulated cells [40]. In addition, PXN is a well-known FA marker [41]. To determine if FAs of cells were affected by FLNa-KD, FA size and number were analyzed in NC and FLNa-KD cells at 1 and 6 h after cell incubation on the glass surface by immunostaining PXN [42], an FA protein. As shown in Fig. 4A, the FAs of NC appears brighter and greater in number compared to NC cells at 1 and 6 h. Both the FA number and size of the FLNa-KD cells were lower than NC cells at 1 and 6 h (Fig. 4B, C), suggesting that FLNa-KD inhibited FA maturation [43]. Previously, it was reported that FA size was negatively related to TFs [44]. Low levels of FLNa expression can increase cancer cell migration by regulating FA assembly and disassembly (FA turnover). The small FA size could be due to the inability of microtubules to extend to the periphery of FLNa-KD cells [45, 46]. Additionally, it was reported that small FAs apply strong TFs to drive cell migration while large FAs anchor the cell to the substrate and produce weak TFs. The results suggest that reduced FA size in the FLNa-KD cells reflects high motility.

### Effect of FLNa-KD on invasion

**Fig. 5:**
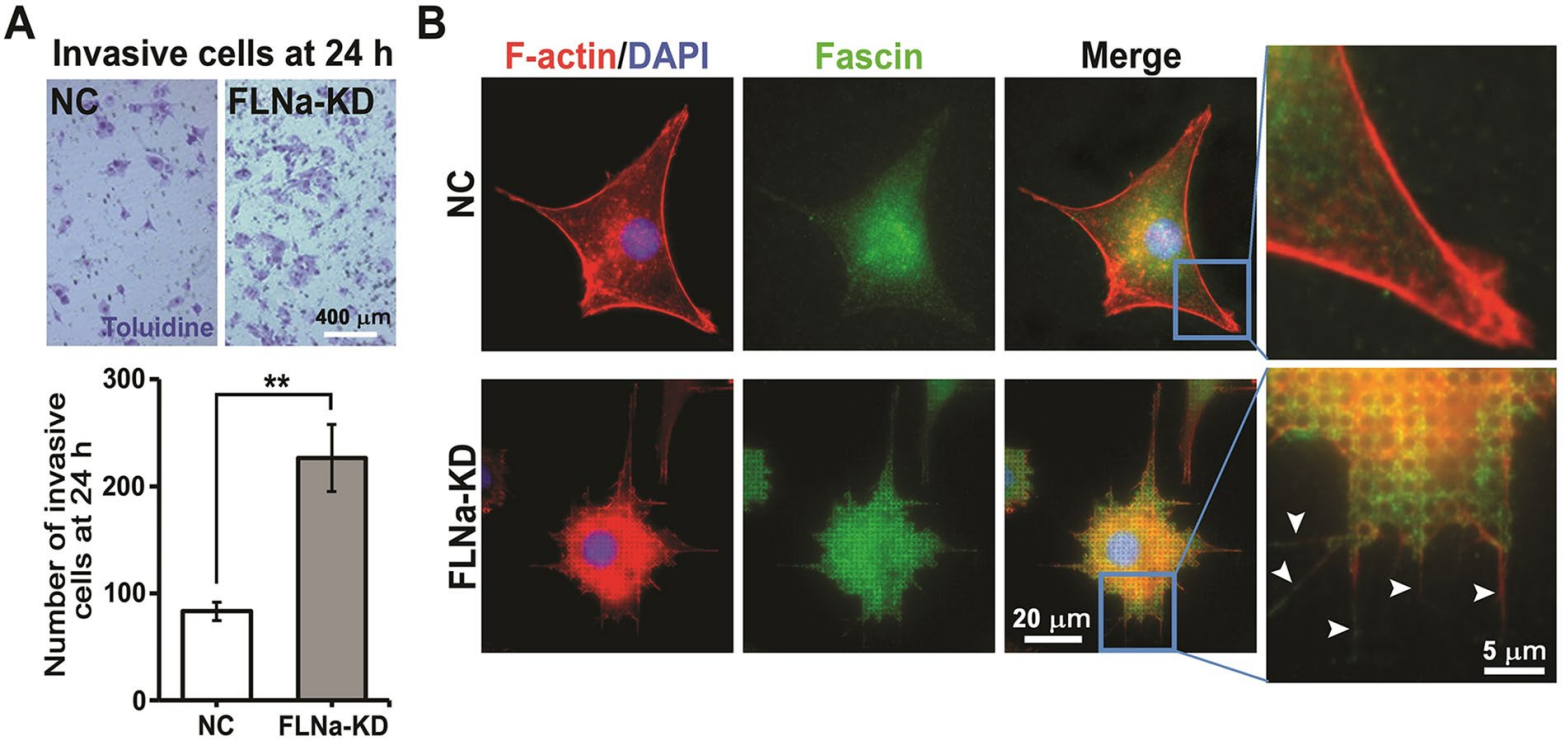
Invasiveness and filopodia formation of NC and FLNa-KD cells. **A** Number of invasive cells obtained from 2 × 10^4^ cells initially seeded on the BioCoat™ Matrigel® Invasion Chamber for 24 h. Each experiment was repeated three times. Error bars represent the mean ± SD of experiments. ** P < 0.01; Student’s t-test. **B** Images of filopodia extension in cells with fluorescent staining of F-actin (RP: red), nuclei (DAPI: blue), and immunofluorescent staining of the actin bundling protein fascin (green)

The effect of FLNa on cell invasion was analyzed using the BioCoat™ Matrigel® Invasion Chamber [47]. Figure 5A shows that FLNa-KD cells are significantly more invasive than NC cells. During invasion, metastatic cells produce thin cellular protrusions called filopodia in the direction of movement to form FAs. Filopodia extensions have strong parallel actin microfilaments that are bundled together by an actin bundling protein called fascin. The immunostaining images of fascin show that FLNa-KD cells displayed a higher number of filopodia extensions than did NC cells on pillars at 1 h (Fig. 5B indicated by white arrows). The difference was not obvious on pillars at 6 h (data not shown). Filopodia are related to cancer invasion. For instance, filopodia inhibition in malignant glioma cells produced a decrease in invasiveness [48]. Taken together, this study showed the migratory ability and invasiveness of FLNa-KD cells using mechanobiological measurements.

## Conclusions

Previous studies showed that FLNa expression or suppression may facilitate or inhibit cancer invasion depending on the cell type, FLNa expression levels, and FLNa interactions with other proteins. Here, TF microscopy using submicron pillars showed that FLNa-KD impairs rigidity sensing and delays cell spreading during the initial phase of cell spreading in U87, while the conventional methods of immunostaining and motility assays showed that the KD facilitated motility and invasion. Our current findings demonstrate that mechanobiological parameters like TF and rigidity sensing can be used to explain the physical mechanisms of highly migratory and invasive properties of metastatic cancer cells.

## Supplementary Information

**Additional file 1: Figure S1.** Rigidity sensing ability of NC and FLNa-KD MDA-MB-231 cells. ** Figure S2. ** Average TF of of NC and FLNa-KD MDA-MB-231 cells after 1 h-incubation on pillars.

**Additional file 2.** NC U87 cells spreading on pillars with the bending stiffness (*k* = 24.2 nN/μm) for 3 min after the cell attached to the top surface of the pillars.

**Additional file 3.** FLNa-KD cells spreading on pillars with the bending stiffness (*k* = 24.2 nN/μm) for 3 min after the cell attached to the top surface of the pillars.

## Data Availability

All data generated or analyzed during this study are included in this published article and its supplementary information files.

## Abbreviations

c-t-c: Center-to-center
CU: Contractile unit
DAPI: 4’,6-diamidino-2-phenylindole
ECM: Extracellular matrix
FA: Focal adhesion
FBS: Fetal bovine serum
FLNa: Filamin a
GAPDH: Glyceraldehyde 3-phosphate dehydrogenase
KD: Knockdown
MEM: Minimum essential medium
NC: Negative control
PXN: Paxillin
PBS: Phosphate-buffered saline
PDMS: Polydimethylsiloxane
RP: Rhodamine phalloidin
RT: Room temperature
siRNA: Small interfering RNA
