# Supplementary material for: Probing mechanobiological role of filamin A in migration and invasion of human U87 glioblastoma cells using submicron soft pillars
Source: Nano Converg. 2021 Jul 2;8:19. doi: 10.1186/s40580-021-00267-6 (PMC8253861; doi:10.1186/s40580-021-00267-6)
Supplement: Supplementary file 1 — Additional file 1: Figure S1. Rigidity sensing ability of NC and FLNa-KD MDA-MB-231 cells. Figure S2. Average TF of of NC and FLNa-KD MDA-MB-231 cells after 1 h-incubation on pillars. [file 40580_2021_267_MOESM1_ESM.docx]

**Probing mechanobiological role of Filamin A in migration and invasion of human U87 glioblastoma cells using submicron soft pillars**

Abdurazak Aman Ketebo^1^, Chanyong Park^1^, Jaewon Kim^1^, Myeongjun Jun^1^, and Sungsu Park^1,2,3*^

^1^Department of Mechanical Engineering, Sungkyunkwan University (SKKU), Suwon 16419, Korea

^2^Department of Biomedical Engineering, Sungkyunkwan University (SKKU), Suwon 16419, Korea

^3^Institute of Quantum Biophysics (IQB), Sungkyunkwan University (SKKU), Suwon 16419, Korea

^*^Corresponding author: Sungsu Park/School of Mechanical Engineering, 2066 Seobu-ro, Sungkyunkwan University (SKKU), Suwon 16419, Korea. E-mail: [nanopark@skku.edu](mailto:nanopark@skku.edu). Tel: +82-31-290 -7431/Fax: +82-31-290-5889. E-mail: [nanopark@skku.edu](mailto:nanopark@skku.edu)


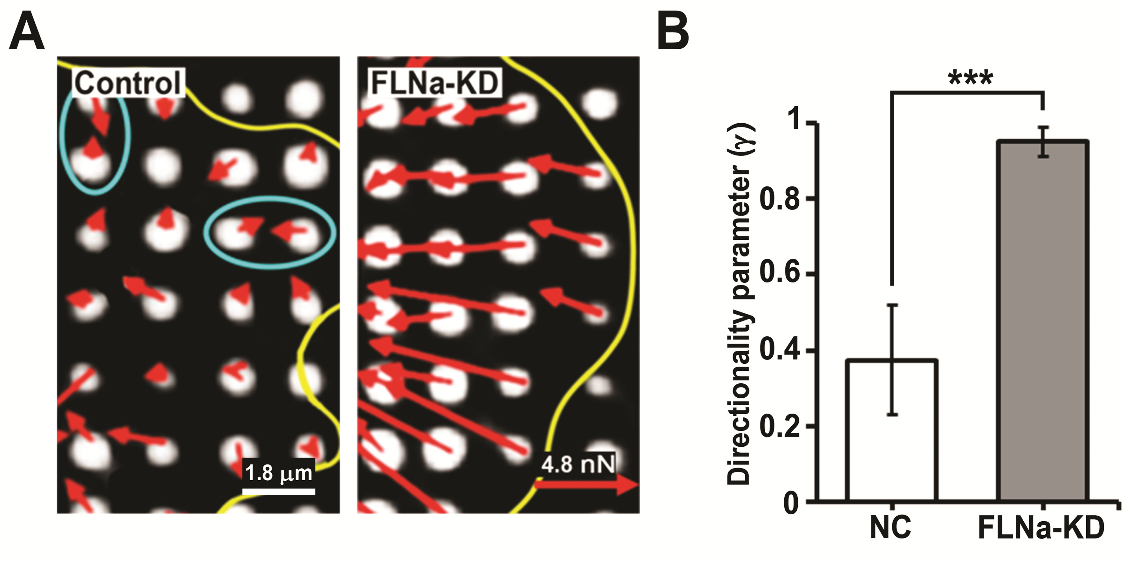


**Fig. S1.** Rigidity sensing ability of NC and FLNa-KD MDA-MB-231 cells. (A) deflection of pillars in NC and FLNa-KD cells near the edge (approximately 34.5 µm^2^) of cells at < 30 min. Local contractions in the NC cells are marked with cyan circles. The red arrows indicate pillar deflection. (B) Directionality parameters (𝛾) of the NC and FLNa-KD cells at < 30 min-incubation. 𝛾 was calculated using the equation presented in the Materials and Methods section. Cell number n = 6; Error bar represents the mean ± SD. *** P < 0.001. Student’s t-test.


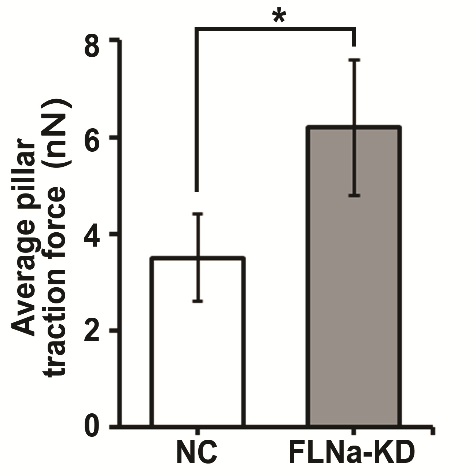
**Fig. S2.** Average TF of of NC and FLNa-KD MDA-MB-231 cells after 1 h-incubation on pillars. Number of samples, (n = 10 cells). Error bar represents the mean ± SD; ** P < 0.05; Student’s t-test.
